# Central network changes in patients with advanced monocular blindness: A voxel‐based morphometric study

**DOI:** 10.1002/brb3.1421

**Published:** 2019-10-01

**Authors:** Wen‐Qing Shi, Yin He, Qing‐Hai Li, Li‐Ying Tang, Biao Li, Qi Lin, You‐Lan Min, Qing Yuan, Pei‐Wen Zhu, Rong‐Bing Liang, Yi Shao

**Affiliations:** ^1^ Department of Ophthalmology Jiangxi Province Ocular Disease Clinical Research Center The First Affiliated Hospital of Nanchang University Nanchang China; ^2^ Department of Ophthalmology Xiang'an Hospital of Xiamen University Fujian Provincial Key Laboratory of Ophthalmology and Visual Science Eye Institute of Xiamen University Xiamen University School of Medicine Xiamen China

**Keywords:** fMRI, monocular blindness, visual functional neuroimaging, voxel‐based morphometry

## Abstract

**Objective:**

To study the changes in gray matter volume (GMV) in patients with advanced monocular blindness (MB) using voxel‐based morphometry (VBM).

**Methods:**

Thirty‐one patients with advanced MB (25 males and six females) and 31 normal controls (25 males and six females) were enrolled. The *t* test was applied to determine the differences in GMV, white matter volume (WMV), and volume of cerebrospinal fluid in different regions of the brain. The local characteristics of spontaneous concentrations of brain tissue were evaluated by the VBM method. The effects of blindness duration on differences in the GMV were evaluated by correlation and regression analyses.

**Results:**

Compared with the control group, the GMV was decreased in the upper right margin, bilateral insular cortex, right cingulate gyrus, left occipital gyrus, and right suboccipital lobe, and negatively correlated with blindness duration in the upper right posterior margin, bilateral insular cortex, and right cingulate cortex.

**Conclusions:**

We found that patients with MB showed abnormal WMV and GMV, as evidenced by local changes in the brain. In addition, reduced GMV in specific parts of the brain was associated with the duration of blindness, which may indicate neuropathological mechanisms of visual loss in patients with MB.

## Graphical abstract

Patients with advanced monocular blindness (MB) showed abnormal white matter volume (WMV) and gray matter volume (GMV), as evidenced by local changes in the brain. In addition, reduced GMV in specific parts of the brain was associated with the duration of blindness, which may indicate neuropathological mechanisms of visual loss in patients with MB.

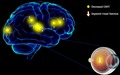

## 1 INTRODUCTION

Blindness is a major public health problem worldwide. According to the World Health Organization, the number of blind people globally reached 36 million in 2015, and 217 million people are affected by moderate to severe visual impairment. The number of blind people is expected to increase to 38.5 million by 2020 and 115 million by 2050 (Bourne et al., 2017). Visual impairment affects the economy and education, reduces quality of life (Eckert et al., 2015), and increases the risk of death (Ramrattan et al., 2001). The health problems caused by blindness are a huge challenge. Blindness can be caused by a variety of eye diseases, such as cataract, glaucoma, and optic neuritis. Blindness can be roughly divided into early blindness and advanced blindness. Currently, surgery and medical treatment are effective ways to treat reversible blindness.

Voxel‐based morphometry of magnetic resonance imaging (MRI) data involves the normalization of all image spaces into the same stereotactic space, and extraction of gray matter (GM), white matter (WM), and cerebrospinal fluid (CSF), smoothing, and, finally, statistical analysis to determine the differences between groups (Thomas & Bahaei, 2012). The voxel‐based morphometry (VBM) technique has been used in ophthalmic diseases such as amblyopia, strabismus, glaucoma, and optic neuritis (Barnes et al., 2010; Huang et al., 2016; Li et al., 2012; Ouyang et al., 2017).

Studies have shown that the functional connectivity (FC) between the primary visual cortex on the right side in the blind and the bilateral auxiliary exercise zone is reduced. In early blind patients, the gray matter volume (GMV) in the early visual area is significantly reduced, and the WM in the optic nerve pathway becomes atrophied. A localized area in the visual cortex of early blindness (EB) exists, in which the reduction in GMV is positively correlated with the duration of blindness (Noppeney, Friston, Ashburner, Frackowiak, & Price, 2005; Pan et al., 2007). The GMV in the visual area of the blind is significantly reduced, whereas apparent GMV is evident in the sensory area (Jiang et al., 2015). Significant changes in mean diffusivity and relative anisotropy have been found in the occipital white matter (WM) of the EB (Shimony et al., 2006). Ptito, Schneider, Paulson, and Kupers (2008) used the VBM technique to study congenital blindness (CB) and found that in contrast to normal controls, the anatomy of the visual system in adults with CB shows significant atrophy in the lateral geniculostriate system, including the optic nerve, optic chiasm, optic radiation, and primary visual cortex. The vision‐related anatomy of patients with CB changes significantly over time, and the related functions are also reconstructed. Subjects with monocular blindness (MB) show FC between the hemispheres in the visual pathway. In addition, the left and right eyes of patients with MB have different functional connections between the cerebral hemispheres (Shao et al., 2018). In the primary visual cortex and higher visual areas of late blindness (LB), the FC between the motor cortex and the somatosensory cortex at rest is reduced, suggesting that visual blindness and visual‐motor function impairment may occur in patients with advanced blindness (Wen et al., 2018). However, whether a difference exists in the vision‐related anatomy and visual cortex between the late blind and the normal control (NC) is not yet clear.

The aim of this study was to further investigate whether a significant structural difference exists in the visual cortex of patients with MB compared to NCs, and how these differences are related to the age at onset and duration of blindness.

## 2 PATIENTS AND METHODS

Experimental group: 31 patients with MB were enrolled at the Department of Ophthalmology, the First Affiliated Hospital of Nanchang University (25 males and 6 females, all of whom were blind in the right eye). The diagnostic criteria for MB were as follows: (a) advanced blindness (18 cases were caused by ocular trauma, and 13 cases were caused by keratitis); (b) normal contralateral eyes, no cataract, glaucoma, optic neuritis, or retinal degeneration. The exclusion criteria were as follows: (a) congenital blindness; (b) impaired visual acuity in the contralateral eye; (c) blindness caused by other eye diseases (e.g., cataract, glaucoma, optic neuritis, macular degeneration, or ocular ischemic disease); (d) history of surgery in both eyes, or long‐term treatment of blindness; (e) mental illness (depression, bipolar disorder, or sleep disorders) or cerebral infarction (cerebral hemorrhage or cerebrovascular malformation); and (f) no obvious abnormalities.

Control group: 31 patients (25 males and 6 females) were selected. Their age, sex, and education level were all matched with participants in the MB group. All NCs met the following criteria: (a) no abnormalities in brain parenchyma on head MRI; (b) uncorrected or corrected visual acuity (VA) > 0.8; (c) no mental disorder; and (d) ability to undergo MRI scans (e.g., no pacemakers or implanted metal devices). All patients and healthy controls have same equivalent education level.

All procedures adhered to the Declaration of Helsinki and recognized the principles of medical ethics. All patients voluntarily participated and were informed of the purpose, methods, and potential risks of the study, and signed informed consent forms.

### 2.1 Structural MRI parameters

Participants were scanned on a 3‐Tesla magnetic resonance scanner (Siemens, Munich, Germany) with a 12‐channel head coil. The fast gradient echo sequence (magnetization prepared rapid acquisition gradient echo, MP RAGE) achieved high resolution covering the whole brain. The T1‐weighted cross‐sectional images were prepared as follows: 176 slices with a thickness of 1.0 mm; echo time of 2.26 ms; repetition time of 1,900 ms; and field of view of 215 × 230 mm. All participants were scanned by the same radiologist, and no abnormalities were observed in the parenchyma of the brain.

### 2.2 Image processing

The MRIcro software (http://www.mricro.com) was used to classify functional data and eliminate incomplete data. The structural images were processed by using a voxel‐based morphometric toolbox on a statistical parameter map (SPM 8) running on the MATLAB 7.9.0. software. The VBM technique is a whole‐brain, unbiased, semi‐automated technique, by which local brain differences in structural magnetic resonance images can be characterized. Individual brains were divided into GM, WM, and CSF, based on VBM8, using the default estimation option (very light deviation regularization; 60 mm truncation for the estimation of Gaussian smoothness of image intensity; and the original European template for affine transformation of the ICBM (International Consortium for Brain Mapping)). Spatial normalization into the Montreal Neuroscience Institute (MNI) standard space was achieved by implementation of the high‐dimensional Dartel (through exponential Lie algebra) method in VBM8. We used the Dartel tool to generate GM and WM templates, and used the generated templates to obtain standardized GM and WM of all participants The volume was then smoothed with a 6‐mm full width at half maximum (FWHM) Gaussian kernel. The normalized, modulated, and smoothed image was submitted to the group‐level analysis.

### 2.3 Processing of data

General linear model analysis was performed using the SPM 8 toolkit. After controlling for age and sex, the differences in GM and WM between patients with MB and healthy controls were observed. The level of statistical significance was set at *p* < .05, and Gaussian random field theory correction was applied (minimum *z* > 2.3). A significant voxel was superimposed on the normalization of 3dt1wi (three‐dimensional magnetization, fast acquisition gradient echo sequence) to generate a color map. Area analysis was performed on adjacent voxels in 20 patients after selecting the voxel threshold.

Statistically significant comparisons were made between the receiver operating characteristic (ROC) curves for each brain region to assess the average redistribution values. The purpose of the correlation analysis was to investigate the relationship between mean outcomes and clinical features in different regions of the brain (*p* < .05).

### 2.4 Correlation analysis

Correlation analysis was used to calculate the relationship between GMV values and duration of blindness in different regions of the brain (*p* < .05 was considered statistically significant).

### 2.5 Analysis of clinical data

Cumulative clinical measurements, including the duration of MB, were recorded and analyzed using independent sample *t* tests.

## 3 RESULTS

### 3.1 Behavioral results

No significant differences in body weight (*p* = .825) or age (*p* = .894) were noted between the MB group and Nc group (*p* > .05). See Table 1 for details.

**Table 1:** Demographics and clinical measurements of MB and NC groups

Condition	MB	NC	*t*	*p*‐value
Male/female	25/6	25/6	N/A	>.99
Age (years)	39.12 ± 9.72	38.76 ± 10.37	0.091	.894
Weight (kg)	58.52 ± 9.26	56.11 ± 12.61	0.083	.825
Handedness	31R	31R	N/A	>.99
Best‐corrected VA‐right eye	0.23 ± 0.11	0.90 ± 0.17	−0.881	.018
Best‐corrected VA‐left eye	0.19 ± 0.09	0.85 ± 0.25	−0.926	.014
IOP‐R (mmHG)	15.23 ± 4.02	16.19 ± 4.92	0.079	.798
IOP‐L (mmHG)	16.15 ± 4.11	16.83 ± 4.56	0.088	.869

Note

Independent *t* tests comparing the two groups (*p* < .05 represented statistically significant differences). Data shown as mean standard deviation or *n*.

Abbreviations: IOP, intraocular pressure; L, left; MB, monocular blindness; N/A, not applicable; NC, normal control; R, right.

### 3.2 Gray and white matter differences

**Figure 1:**
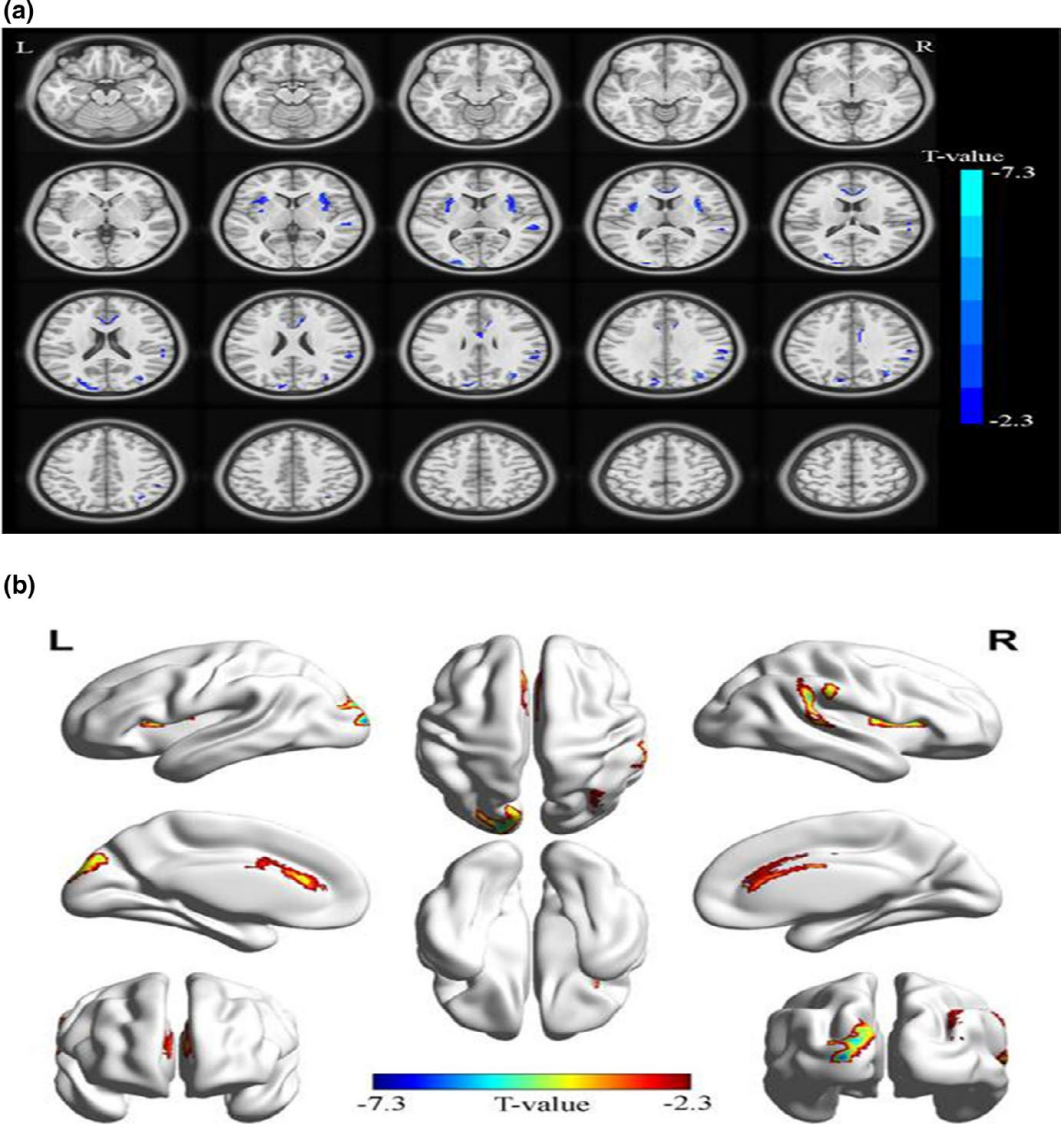
Spontaneous brain activity in the MB versus NC. (a, b) Compared with the NC group, the GMV was reduced in the right margin of the right side in the patients with MB, as well as the bilateral insular lobe, the right anterior cingulate gyrus, the left occipital gyrus, and the right suboccipital lobe. L, left; MB, monocular blindness; NC, normal control; R, right

**Figure 2:**
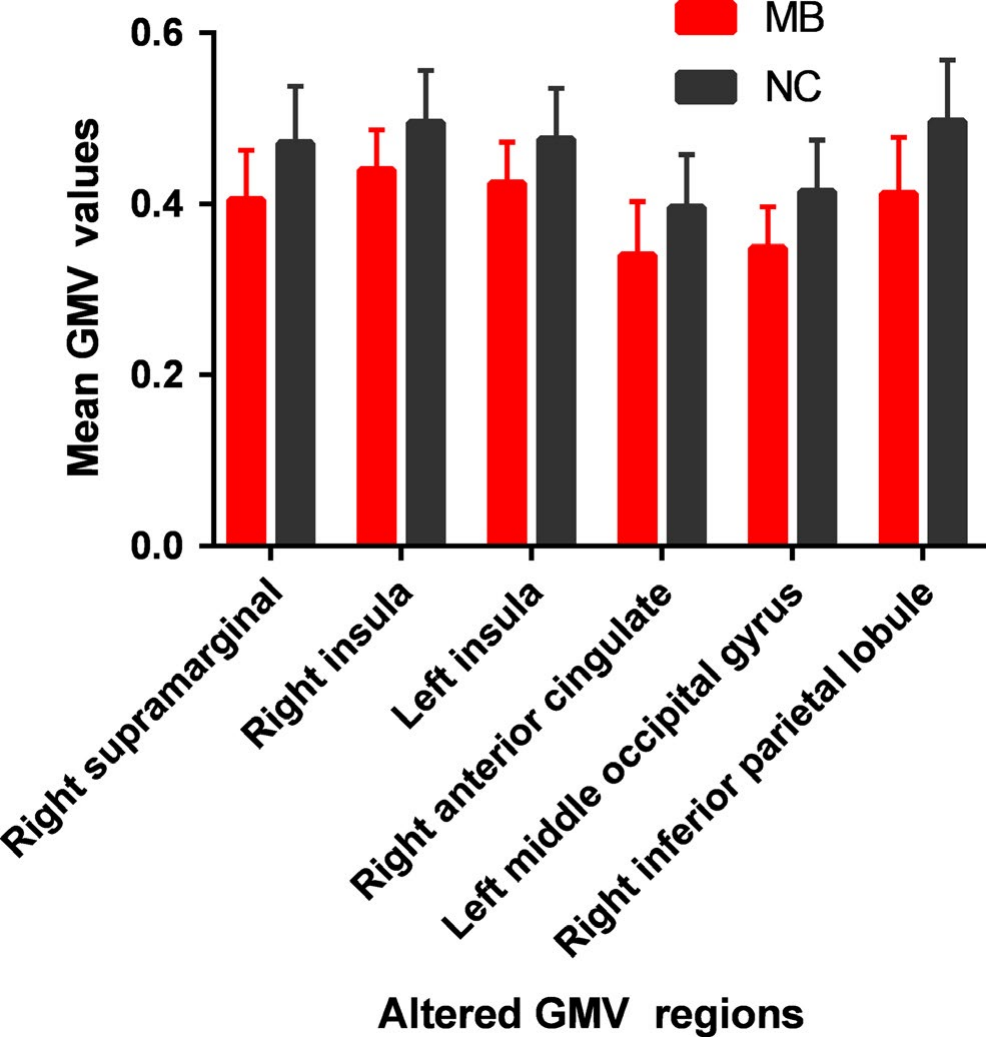
The mean GMV. values between the MB and NC group. GMV, gray matter volume; MB, monocular blindness; NC, normal control

Compared with the NC group, the GMV was reduced in the right margin of the right side in the patients with MB, as well as the bilateral insular lobe, the right anterior cingulate gyrus, the left occipital gyrus, and the right suboccipital lobe. See Figure 1 and Table 2 for details. The mean GMV values between the MB group and NC group are shown in Figure 2. We also compared the whole‐brain GMV, WMV, and CSF volume in the MB group with those in the NC group. See Table 3 for details. No significant differences were noted between the MB group and NC group (*p* > .05).

**Table 2:** GMV differences between MB and NC groups

Comparison	BA	MNI coordinates			Cluste*r* size	Peak *t*‐value
		*X*	*Y*	*Z*		
MB < NC						
RSM	40	51	−30	6	642	−5.870
RI	13	33	−1.5	12	537	−7.344
LI	13	−3.45	1.5	15	413	−7.115
RAC	24	10.5	27	22.5	496	−5.258
LMOG	18/19	−18	−99	7.5	555	−6.238
RIPL	40	36	−72	31.5	342	−6.229

Abbreviations: BA, Brodmann area; GMV, gray matter volume; LI, left insula; LMOG, left middle occipital gyrus; MB, monocular blindness; MNI, Montreal Neurological Institute; NC, normal control; RAC, right anterior cingulate; RI, right insula; RIPL, right inferior parietal lobe; RSM, right supra marginal.

**Table 3:** Comparison of total brain volume between MB and NC groups

	WMV	CSF (ml)	CSF (ml)	GMV (%)	WMV (%)
MB	479.64 ± 68.52	346.42 ± 76.09	1,482.08 ± 191.58	44.33 ± 35.61	32.37 ± 18.24
NC	490.77 ± 46.48	326.36 ± 48.35	1,494.46 ± 128.33	45.22 ± 28.83	32.60 ± 18.19
*t*	−0.761	1.259	−0.304	−1.1	−1.07
*p*‐value*	.45	.213	.762	.276	.289

Abbreviations: CSF, cerebrospinal fluid; GMV, gray matter volume; MB, monocular blindness; NC, normal control; WMV, white matter volume; WMV, white matter volume.

* *p* < .05, independent *t* tests comparing two groups; the data are shown in mean ± standard deviation.

### 3.3 Correlation analysis

**Figure 3:**
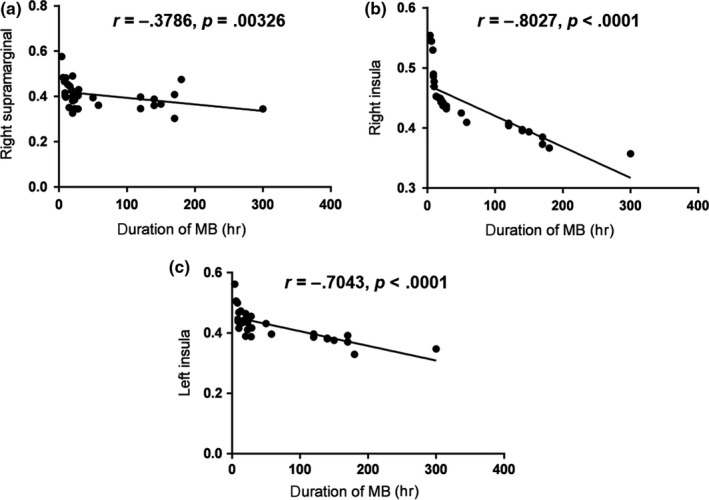
Correlations between the mean GMV and duration of blindness in the different brain areas. (a) The GMV value of the upper right margin of the MB group was negatively correlated with the duration of blindness (*r* = −.3786, *p* = .0326). (b) The GMV value of the right insular cortex of the MB group was negatively correlated with the duration of blindness (*r* = −.8027, *p* < .0001). (c) The GMV value of the left insular cortex of the MB group was negatively correlated with the duration of blindness (*r* = −.7043, *p* < .0001)

Correlations between the mean GMV of different brain regions and duration of blindness yielded the following results. The GMV value of the upper right margin of the MB group was negatively correlated with the duration of blindness (*r* = −.3786, *p* = .00326). Furthermore, the GMV value of the right insular cortex of the MB group was negatively correlated with the duration of blindness (*r* = −.8027, *p* < .0001). Similarly, the GMV value of the left insular cortex of the MB group was negatively correlated with the duration of blindness (*r* = −.7043, *p* < .0001). See Figure 3 for details.

### 3.4 Receiver operating characteristic curves

**Figure 4:**
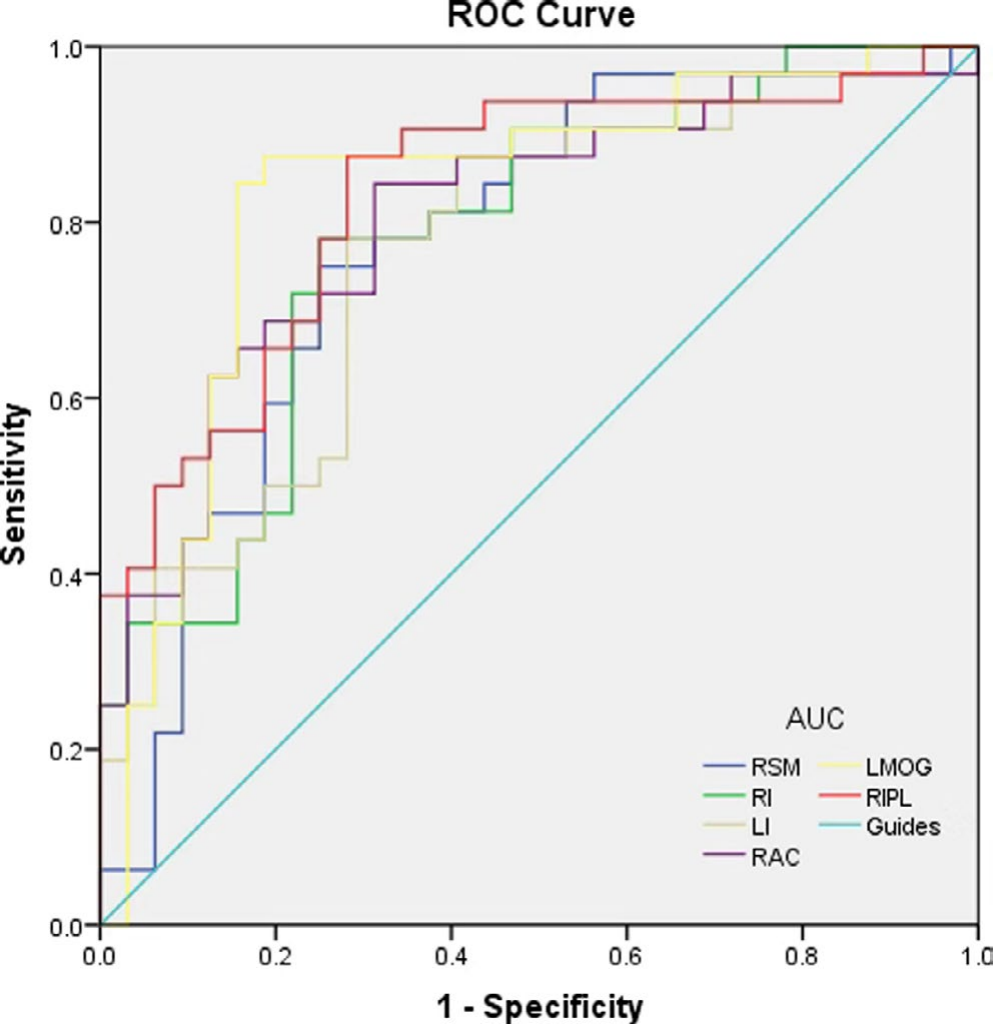
ROC curve analysis of the mean GMV values for altered brain regions. The average GMV values of different brain regions were analyzed using ROC curves. The area under the curve for the FC values were as follows: 0.774, (*p* < .001; 95% CI: 0.657–0.892) for RSM; RI 0.782 (*p* < .001; 95% CI: 0.670–0.895); LI 0.768 (*p* < .001; 95% CI: 0.652–0.884); RAC 0.804 (*p* < .001; 95% CI: 0.695–0.913); LOMG 0.831 (*p* > .05; 95% CI: 0.723–0.939); and RIPL 0.832 (*p* < .001; 95% CI: 0.731–0.933). AUC, area under the curve; GMV, gray matter volume; LI, left insula; LMOG, left middle occipital gyrus; RAC, right anterior cingulate; RI, right insula; RIPL, right inferior parietal lobe; ROC, receiver operating characteristic; RSM, right supra marginal

The average GMV values of different brain regions were analyzed using ROC curves. The area under the curve for the GMV values were as follows: 0.774, (*p* < .001; 95% CI: 0.657–0.892) for right supramarginal gyrus; RI (right insular) 0.782 (*p* < .001; 95% CI: 0.670–0.895); LI 0.768 (*p* < .001; 95% CI: 0.652–0.884); right anterior cingulate gyrus 0.804 (*p* < .001; 95% CI: 0.695–0.913); left middle occipital gyrus 0.831 (*p* > .05; 95% CI: 0.723–0.939); and right inferior parietal lobe 0.832 (*p* < .001; 95% CI: 0.731–0.933). See Figure 4 for details.

## 4 DISCUSSION

**Figure 5:**
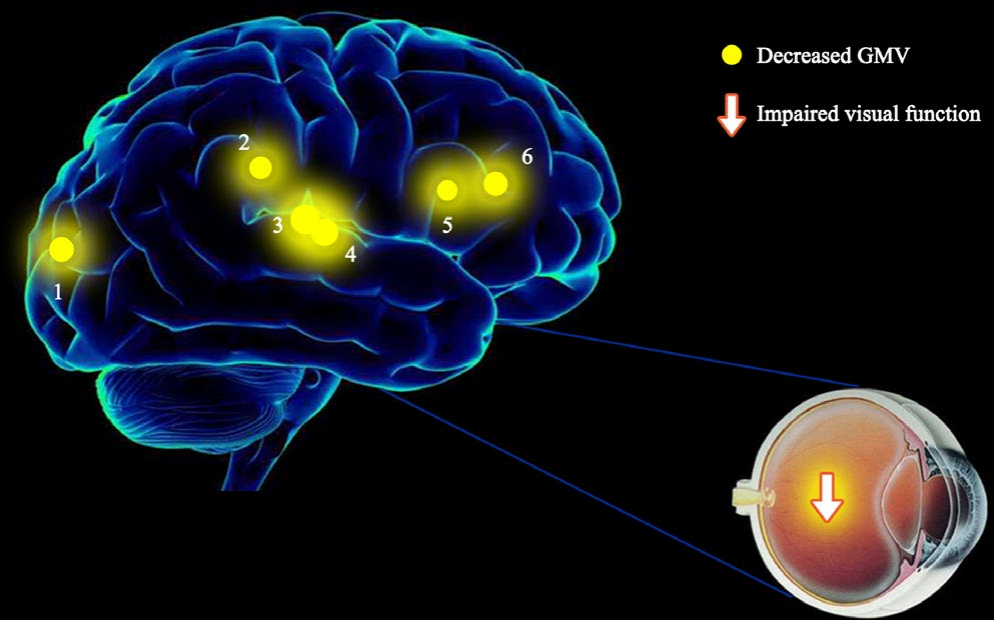
The mean GMV values of altered brain regions in the MB group. Compared with the NC, the GMV values of the following regions were reduced to various extents: 1—right insula (BA 13, *t* = −7.344), 2—left insula (BA 13, *t* = −7.115), 3—left middle occipital gyrus (BA 18/19, *t* = −6.238), 4—right inferior parietal lobe (BA 40, *t* = −6.229), 5—right supra marginal (BA 40, *t* = −5.870), and 6—right anterior cingulate (BA 24, *t* = −5.258)

To the best of our knowledge, our study is the first to use the VBM method to study changes in the GMV of patients with MB. The GMV in the right margin, bilateral insular cortex, right anterior cingulate gyrus, left occipital gyrus, and right suboccipital lobe were all reduced in the patients with MB (Figure 5).

Studies have shown that blindness may enhance nonvisual sensory perception and cognition (i.e., compensatory effects), such as auditory (Hötting & Röder, 2009), tactile (Lewis, Saenz, & Fine, 2010), and chemosensory tasks (Kupers & Ptito, 2014). The supramarginal gyrus (SMG) is a speech processing network and an important component (Oberhuber et al., 2016) associated with visual word recognition (Stoeckel, Gough, Watkins, & Devlin, 2009) and oral working memory (Deschamps, Baum, & Gracco, 2014). A previous study has shown that the SMG is activated when healthy participants made speech word decisions (Hartwigsen et al., 2010). In addition, patients with blindness have shown enhanced SMG functional connectivity (Heine et al., 2015). Li et al. (2016) found that patients with MB have elevated SMG amplitude of low‐frequency fluctuation (ALFF) values on the left side, and MB was unable to activate the SMG. Therefore, we speculated that the brain coding function of speech coding and processing is a compensatory mechanism for the loss of visual acuity in patients with MB. In our study, the GMV of the right margin of the patients with MB was decreased, and the GMV value of the right margin in the MB group was negatively correlated with the disease course (*r* = −.3786, *p* = .0326). Thus, we further confirmed that MB may cause dysfunction of the SMG.

The insular cortex participates in emotional processing (Herwig et al., 2010; Surguladze et al., 2010), and their dysfunction leads to the abnormal subjective sensory states of multiple mental disorders. For example, structural and functional defects in the insula are associated with anxiety (Stein, Simmons, Feinstein, & Paulus, 2007; Terasawa, Shibata, Moriguchi, & Umeda, 2013) and are involved in disorders of emotional processing in patients with schizophrenia (Wylie & Tregellas, 2010). The GM of the insular cortex in patients with major depression is significantly reduced (Stratmann et al., 2014; Takahashi et al., 2010). A previous study found that voxel‐mirrored homotopic connectivity values were increased in the insular cortex of blind patients (Shao et al., 2018). In the present study, the GMV in bilateral insular cortex of late‐stage blindness was decreased and that in the bilateral insular lobe was negatively correlated with the duration of blindness. Thus, MB may also cause emotional disorders.

The occipital lobe is the area of visual processing in the brain. Studies have confirmed that blindness can cause changes in the functions of the occipital cortex that are responsible for vision. Functional magnetic resonance imaging and positron emission tomography studies have determined that the medial region of the occipital bone responds to various stimuli and activities (Renier et al., 2010). The occipital lobe is part of the visual cortex involved with spatial processing (Tu, Qiu, Martens, & Qinglin, 2013) and category‐selective attention (Bavelier & Neville, 2002). In one study of congenital blindness, changes occurred in occipital cortical function to support nonvisual perception and cognitive function (Jiang et al., 2015). Studies have found that blind people usually have significantly high regional homogeneity (ReHo) in primary and higher visual pathways (Büchel, 1998), including the left occipital bone (POS), right calcarine sulcus, right middle occipital gyrus, and right superior occipital gyrus. Early blind people also show selective attentional augmentation, which may help to improve auditory perception (Elbert et al., 2002). Most studies of cross‐model plasticity caused by blindness have been performed in individuals who were initially deprived of vision. Auditory or tactile recruitment of the occipital region has been demonstrated in individuals with advanced blindness (Büchel, 1998; Burton, 2003; Voss, Gougoux, Lassonde, Zatorre, & Lepore, 2006). In the present study, the GMV of the occipital gyrus was decreased in advanced blindness, and no correlation was observed with the duration of blindness. Therefore, we speculated that occipital dysfunction was present in the subjects with late blindness.

The parietal lobe is involved in a variety of advanced cognitive functions that support the higher functions unique to humans, such as mathematics cognition, the semantic and pragmatic aspects of language, and abstract thinking. In a previous fMRI study, the inferior parietal lobes were activated in episodic memory (Cabeza, Ciaramelli, Olson, & Moscovitch, 2008), working memory, and perception of three‐dimensional objects (Grefkes & Fink, 2005; Tsutsui, Jiang, Sakata, & Taira, 2003). In the present study, the GMV of the lower right parietal lobe was decreased, which may reflect functional reorganization to compensate for impaired visual acuity in patients with MB.

The anterior cingulate cortex (ACC) is part of the limbic system and the key center for the processing of emotional pain. It participates in many cognitive and emotional processes, such as attention (Bryden, Johnson, Tobia, Kashtelyan, & Roesch, 2011; Luks, Simpson, Feiwell, & Miller, 2002), decision (Blanchard, Strait, & Hayden, 2015; Lapish, Durstewitz, Chandler, & Seamans, 2008), and pain management (Urien, Xiao, & Dale, 2018). A previous study showed that the right dorsal ACC may be related to visual cognition and emotion (Shinoura, Yamada, & Tabei, 2013). Huang et al. (2017) found that the ReHo value of the ACC on the right side of patients with MB was significantly reduced, indicating the disturbance of synchronous nerve activity in patients with MB. In the present study, the decline in GMV in the right ACC in the MB group further confirmed this conclusion.

## 5 CONCLUSION

We found that patients with MB have abnormal WMV and GMV involvement in regional brain changes, and the reduction of GMV in partial regions of the brain is associated with the duration of blindness. These findings may indicate the neuropathological mechanism of visual loss in patients with MB. In addition, changes in these brain activities can also be used as valuable clinical indicators. However, our study also has some limitations, such as the relatively small size of the sample. In addition, the inclusion criteria for MB were not strict, such as blindness in either the left or right eye, which may have affected the accuracy of the results. During the scanning period, some subjects may have been involved in physical activities, which may have affected the results. In future research, we will use a variety of techniques to study the neuropathological changes that occur in patients with MB.

## CONFLICT OF INTEREST

The authors declare no competing interests.

## ETHICAL APPROVAL AND INFORMED CONSENT

This study was performed under the approval of the Medical Research Ethics Committee of the First Affiliated Hospital of Nanchang University. The methods in this study were conducted under relevant guidelines and regulations. All participants involved were offered the whole study design and signed the informed consent.

## Data Availability

The datasets generated during and/or analyzed during the current study are available from the corresponding author on reasonable request.
